# Using poetry to elicit internal medicine residents’ perspectives on wellness

**DOI:** 10.1136/postgradmedj-2021-141493

**Published:** 2022-04-04

**Authors:** Larissa E Wietlisbach, David A Asch, Whitney Eriksen, Frances K Barg, Lisa M Bellini, Sanjay V Desai, Abdul-Rakeem Yakubu, Judy A Shea

**Affiliations:** Medicine, Perelman School of Medicine, University of Pennsylvania, Philadelphia, Pennsylvania, USA; Medicine, Perelman School of Medicine, University of Pennsylvania, Philadelphia, Pennsylvania, USA; Medicine, Perelman School of Medicine, University of Pennsylvania, Philadelphia, Pennsylvania, USA; Medicine, Perelman School of Medicine, University of Pennsylvania, Philadelphia, Pennsylvania, USA; Medicine, Perelman School of Medicine, University of Pennsylvania, Philadelphia, Pennsylvania, USA; Medicine, Johns Hopkins University, Baltimore, Maryland, USA; Medicine, Perelman School of Medicine, University of Pennsylvania, Philadelphia, Pennsylvania, USA; Medicine, Perelman School of Medicine, University of Pennsylvania, Philadelphia, Pennsylvania, USA

**Keywords:** medical education & training

## Abstract

**Purpose:**

To elicit internal medicine residents’ perspectives on wellness through poetry writing, examining (1) response rates, (2) the tone/sentiment of their submissions and (3) the primary thematic content.

**Study design:**

In academic year 2019–2020, a random sample of 88 residents from four internal medicine residency programmes was invited to participate in a year-long study of wellness. In December 2019, an open-ended prompt asked residents to write a poem reflecting on their well-being. Responses were inductively coded using content analysis techniques.

**Results:**

The response rate for the poetry prompt was 94%. The tone of the entries was most often neutral or contradictory (42%), followed by negative (33%) and positive (25%). There were three main themes: (1) Mindsets: most residents simply wanted to make it through their programme; (2) wellness influencers: the main wellness supporters were external to the programme such as vacationing and exercise; within hospitals, friendships with colleagues and boosted wellness and (3) scheduling/repetition: difficult schedules drained energy as did the monotony of administrative tasks.

**Conclusions:**

Poetry appears to be an innovative and effective vehicle to elicit residents’ perspectives without compromising response rate. Poetry survey techniques allow medical trainees to provide powerful messaging to leadership. Most of what is known about trainee wellness is derived from quantitative surveys. This study showed medicine trainees’ willingness to engage in poetry and add richness and personal detail to highlight key drivers of wellness. Such information provides context and brings attention in a compelling manner to an important topic.

Going, leaving, here

Every day it feels the same

I am surviving.

Resident 205, PGY1, Program 2

## Introduction

In the past two decades, literally hundreds of studies have been published documenting the stress encountered by healthcare professionals [1–4]. Moreover, across medical specialties and multiple constructs, physician residents appear to be less well off than fellows and medical students [5, 6]. The quantitative estimates of emotional and psychological health states have been the backbone in setting the stage to design interventions that improve well-being [7]. We wondered if another type of data, specifically poetry, might give insights into wellness and well-being that would add context to the numbers.

The idea of poetry in medicine is not new. Nearly 20 years ago, educators asked if and how poetry could be used to make better doctors [8]. Narrative medicine and visual art instruction rotations and classes have proliferated [9, 10]. There is also some evidence that practitioners believe poetry enhances personal wellness [11, 12]. However, we were not looking at the impact of poetry creation on wellness per se, but rather the usefulness of poetry as a data collection tool that enables collection of rich, illustrative portrayals of how medical training intersects with wellness.

In the current study, we explored if and how internal medicine residents responded when asked to write a poem on their well-being. During academic year 2019–2020, internal medicine trainees received biweekly surveys with prompts on different aspects of wellness [13]. One of these prompts asked residents to write a poem (a haiku or a limerick) describing their well-being. Our intentions with this prompt were threefold: assess resident willingness to engage in artistic endeavours through response rate and explore the tone and the thematic content of the responses.

## Methods

All 396 internal medicine residents from four residency programmes were invited to participate in a survey-based study throughout academic year 2019–2020 [13], 186 volunteered. A random sample stratified by programme and training levels resulted in 88 consented residents. Participants received 18 open-ended survey prompts, one approximately every 2 weeks, from October 2019 to May 2020. A $100 Amazon gift card was delivered for every three completed responses.

On 9 Decembe 2019, the poetry-based prompt instructed residents as follows:

For this survey, we would like for you to get creative! Please compose a haiku or a limerick (G-Rated) to reflect on your well-being. Below, select which poem you would rather compose, and a box will appear with examples and a link to learn about each type of poem.

Two members of the study team coded the entries for the poetry prompt using content analysis techniques [14, 15]. The process started with independent reading of the entries and then a discussion of initial coding strategies. The initial intent was to align the content of the poems according to the Shanafelt and Noseworthy framework: individual, work unit, organisation and national [7]. This framework clarifies that causes of workplace burnout and solutions for wellness strategies can be situated at many levels and argues for organisations to take a lead in effecting solutions. One coder attempted to independently apply the initial coding structure. However, the overarching framework did not fit well with the relatively focused probe. Ultimately, each poem was coded for tone (positive, negative or neutral/contradictory) and content. The coders worked independently and met to discuss discrepancies and arrive at consensus. Agreement was above 80% for tone and content codes and subcodes. The majority of the poems touched on multiple themes.

## Results

### Participation

Residents detailed their perspectives of their residency programmes through their haikus and limericks. The response rate was 94%. Overall, 70% of the entries were Haikus; 15% were Limericks and 15% were other forms of poetry.

### Tone

Overall, the tone of the poems was most often neutral or contradictory (42%), followed by negative (33%) and positive (25%).

The modal group of responses was contradictory, including both positive and negative sentiments.

Low expectations

Meeting them almost daily

Working to improve.

Resident 103, PGY1, Program 1

#### Negative tones

Negative tones typically reflected residents exhaustion, their sense of being overwhelmed and their doubts of wellness efforts. Some poems highlighted the monotony of residents’ days within the hospital and clinics.

The exhaustion stings

In the unit time flies by

No rest for the weak.

Resident 312, PGY2, Program 3

A handful of residents used their poetry to express positive themes—optimism, their call to medicine and appreciation for coworkers.

With coffee in hand

I calmly take on the day

Oh what a good day.

Resident 215, PGY2, Program 2

### Content

Content analysis revealed themes that fell into three main categories—mindset, wellness influencers and scheduling/repetition. Additional exemplary quotes from each of these themes are shown in Table 1 with shading and italics indicating the positive, negative or neutral/contradictory tone(s) of the poems.

**Table 1:** Representative poems from internal medicine residents describing wellness*

		Quote 1	Quote 2
Mindset	Surviving	*Smile,dodge,smile,* *Control the bile*. *Lead to a save,* *Have everyone wave*. *Smile,dodge,smile.* *Resident 411, PGY2, Program 4*	*Stretched to my limit* *Just wanting to do my best* *Everyone agrees.* *Resident 412, PGY2, Program 4*
	Thriving	*Intern year is fun* *So many awesome new friends* *But I am so dumb.* *Resident 406, PGY1, Program 4*	Grey is the day now A million suns are within Ours is now to shine. Resident 116, PGY2, Program 1
Wellness	Outside hospital	Leaving from my work Going for a run, breathe free Leaving stress behind. Resident 408, PGY1, Program 4	Sleep, run, read, wine, eat Keep me wholesome day by day And then we repeat. Resident 302, PGY1, Program 3
	Within hospital	Breakfast with my team The best way to end night float It’s the little things. Resident 108, PGY1, Program 1	*“I'll be here all night”* *“So will I”, the patient laughs* *Both thinking of home.* *Resident 105, PGY1, Program 1*
	Barriers	In our hospital, We admit, discharge, admit; Learning by volume. Resident 222, PGY3, Program2	*Wellness time gets filled* *With what it is hard to say* *Then it’s back to work.* *Resident 114, PGY2, Program1*
	Environment	Bright and shiny day No ICU noise to hear Just chirping of birds. Resident 303, PGY1, Program 3	*Watching patients in the ICU suffer,* *I find myself needing a buffer*. *Some sun and fresh air are my preferred self^-^care, but as the days grow shorter, it gets tougher.* *Resident 306, PGY1, Program 3*
Repetition/schedule	Repetition	Wake, Sign in, Pre^-^round Round, Round, Order, Write, Sign out Nothing matters here. Resident 401, PGY1, Program 4	*I am very tired* *Tomorrow will be better* *Each day is the same.* *Resident 305, PGY1, Program 3*
	Schedule	80 hours per week, With little time for sleep. And they wonder about, “What’s this burnout?” 80 hours per week. Resident 223, PGY3, Program 2	Another twenty^-^ eight hour call. Keeping patients safe, but not myself. Resident 204, PGY1, Program 2
	Exhaustion	Long call with no sleep Pushing limits of my brain Now sole task of rest. Resident 216, PGY2, Program 2	*My body is tired* *But my mind is wired* *The clock strikes five* *I'm alive* *But my body is tired.* *Resident 109, PGY2, Program 1*

*Each poem’s tone is signified by the shading of its cell—where negative tone is grey, positive tone is white and the neutral/contradictory statements are in italics.

#### Mindset

The first main theme was one of mindset. Within this theme were many poems about survival. Through the monotony and exhausting schedules, many residents aimed to simply make it through their day/programme. There were poems illustrating efforts to try one’s best, while acknowledging that their best may only reach the baseline expectations. Forward-looking thoughts acknowledged that the arduous process of residency training would ultimately help them be more knowledgeable, efficient and capable of serving their patients.

As I drive to work

I dread pre-rounding this morn

Just survive I say.

Resident 407, PGY1, Program 4

However, not all residents viewed themselves as simply surviving—some were also thriving and really enjoying their time pursuing their dreams, calls to medicine and getting to push themselves in terms of learning and making new friends in their colleagues.

Caring and healing,

This is a dream job each day,

So much left to learn.

Resident 203, PGY1, Program 2

#### Wellness influencers

When residents spoke of wellness, they nearly exclusively mentioned wellness supporters outside of the hospital and clinics; residents achieved stress relief from yoga, meditation and spending time with pets. Also, many residents highlighted how planning and going on vacation brought a sense of wellness.

Taking time to read,

chat, breath[e], and do yoga

always calms me.

Resident 112, PGY2, Program 1

When describing wellness supporters within the residency programmes and hospitals, emphasis was put onto the joy and fulfilment that come from interacting with colleagues.

Together we work

Growing in love and laughter

Residency friends.

Resident 316, PGY3, Program 3

However, some residents described barriers to wellness within their programmes, expressing disagreement with how programmes highlight well-intentioned wellness initiatives rather than provide time off and better compensation.

Wellness is less difficult than it seems,

Maslow’s hierarchy has the right themes,

We need time off for other parts of life,

A sense of community to process the strife,

And for personal health, the right means.

Resident 320, PGY3, Program 3

Interestingly, a factor that impacted resident wellness within and outside of the workplace was the residents’ environment, including access to fresh air, natural light and crowding of work rooms.

As I enter, dark skies

False lights cruel to my worn eyes

Til I leave, dusk past.

Resident 402, PGY1, Program 4

#### Scheduling/repetition

The last theme was scheduling/repetition. Residents painted the day-to-day as a dreary, monotonous landscape consisting of repetition: administrative work, unfulfilling time in front of the computer and the tedious cyclical nature of resident responsibilities.

Relentless ringing

The phone calls just do not stop

A longing for peace.

Resident 118, PGY3, Program 1

Compounding the repetitive nature of residency, schedules were reported to be rigorous and tiring. Residents often tied the long shifts to a sense of being overwhelmed and exhausted.

Girl on night float is so tired

Doesn't care if she sleeps in and gets fired

Eyes are red

Missing my bed

Girl on night float is so tired.

Resident 410, PGY2, Program 4

## Discussion

Quantifying and improving healthcare workers’ wellness has been an important part of medical education literature for two decades. We explored a unique research tool in the academic medical space: the use of poetry in survey prompts to internal medicine residents. We found that requesting poetry did not deter residents from responding, yielding a higher response rate than what is often seen [16]. Some submissions were decidedly positive or negative, but more were neutral or contradictory in tone.

Similar to existing work that calls for the addition of humanities to medical education [9, 17, 18], we believe the poetry allowed a connection between the authors (the residents) and their audience that would not have been possible with traditional closed-ended survey-based methods. Inviting and allowing for creativity provided an opportunity for residents to look inward—and outward—and express feelings, both positive and negative. A poetry-based survey provided space for residents to engage with self-reflection, mindfulness and actualisation of their opinions. Arguably, by providing illustrative context to the burnout statistics among medical trainees, the content revealed in the form of art may be more impactful in communicating key messages to programme leaders compared with more traditional metrics of wellness. Interestingly, unlike prior work that has analysed creative projects [19], we did not find many of the poems centred on patients. Patients were mentioned, to be sure, but it was almost as if they were in the background. Perhaps this is a result of the specificity of the prompt asking residents to focus on their own well-being.

This study is limited by its single survey prompt with limited scope. We chose the format of haikus and limericks to be mindful of the residents’ limited time. Many of our respondents used other poetic forms, but all responses were short. Moreover, as mentioned above, the prompt’s focus on residents’ own wellness may have led to the limited mention of patients and their relationships with them, unlike prior work with poignant stories of patients [13, 19]. Second, we sampled within just four internal medicine programmes in the Northeast USA. Broader sampling might have yielded different results. Third, the prompt was delivered in December. The multiple mentions of limited hours of lightness remind us that wellness is linked to external conditions. Fourth, the data were collected as part of a research study with financial incentives, and thus the response rate may not reflect what would occur in practice. In fact, there is a sizeable literature that supports the value of incentives in increasing response rates [20]. 

We were encouraged by the high response rate and rich thematic content elicited from the poetry prompt. The idea of linking poetry to medicine is decades old [21], but our results suggest similar exercises may be a useful tool in current efforts to build a culture of wellness [5]. The themes and subthemes drawn from the poems, as well as the poems themselves, can bring attention in a compelling manner to an important topic. In the near future it will be important to disseminate these results to educational programme leadership who have the authority and responsibility to create training environments that support trainee wellness—as called to by many of the trainees’ poems. As mentioned above, we believe these poems may provide context in the call for a stronger emphasis on wellness in medical training. A second audience for these results is the faculty who direct increasingly popular narrative medicine curricula; these results can inspire and guide future initiatives. Adding another tool for learner expression should be encouraged. Anecdotally, at least some of the trainees found the task enjoyable and welcomed the creative outlet. Finally, methodologically, it will be useful to ask some ‘value added’ questions and learn if the arts can add to the respondent’s experience in a way that is unique when compared with other forms of expression.

Main messages

- Internal medicine residents readily responded to invitations to write poetry about their wellness.
- The tone of the poetry often combined positive and negative sentiments.
- The positive ‘wellness’ content of the poetry most often referenced activities and people outside of the hospital.
- The negative or ‘less well’ content of the poetry suggested many aspects of the training structure that might be targets for interventions.

Current research questions

- Will medicine trainees even respond to requests to write poems about wellness?
- What is the tone and content of the poems?

What is already known on the subject

- Many healthcare workers experience high levels of stress and burnout.
- Poetry and other art forms are often introduced into curricula as a means to increase well-being and awareness.

## Data Availability

No data are available.
